# The predictive value of left ventricular global longitudinal strain in normotensive critically ill septic patients

**DOI:** 10.5935/2965-2774.20230378-en

**Published:** 2023

**Authors:** Timor Omar, Kamil İnci, Yusuf Oflu, Mustafa Dilek, Zeynep Binici Çelik, Soner Kına, Doğan İliş, Halil Murat Bucak

**Affiliations:** 1 Department of Cardiology, Faculty of Medicine, Kafkas University - Kars, Turkey; 2 Kars Harakani State Hospital - Kars, Turkey

**Keywords:** Sepsis, Ventricular dysfunction, Mortality, Echocardiography, Critical care, Global longitudinal strain

## Abstract

**Objective:**

Evaluation of left ventricular systolic function using speckle tracking echocardiography is more sensitive than conventional echocardiographic measurement in detecting subtle left ventricular dysfunction in septic patients. Our purpose was to investigate the predictive significance of left ventricular global longitudinal strain in normotensive septic intensive care patients.

**Methods:**

This observational, prospective cohort study included septic normotensive adults admitted to the intensive care unit between June 1, 2021, and August 31, 2021. Left ventricular systolic function was measured using speckle-tracking echocardiography within 24 hours of admission.

**Results:**

One hundred fifty-two patients were enrolled. The intensive care unit mortality rate was 27%. Left ventricular global longitudinal strain was less negative, which indicated worse left ventricular function in non-survivors than survivors (median [interquartile range], -15.2 [-17.2 - -12.5] *versus* -17.3 [-18.8 - -15.5]; p < 0.001). The optimal cutoff value for left ventricular global longitudinal strain was -17% in predicting intensive care unit mortality (area under the curve, 0.728). Patients with left ventricular global longitudinal strain > -17% (less negative than -17%, which indicated worse left ventricular function) showed a significantly higher mortality rate (39.2% *versus* 13.7%; p < 0.001). According to multivariate analysis, left ventricular global longitudinal strain was an independent predictor of intensive care unit mortality [OR (95%CI), 1.326 (1.038 - 1.693); p = 0.024], along with invasive mechanical ventilation and Glasgow coma scale, APACHE II, and SOFA risk scores.

**Conclusion:**

Impaired left ventricular global longitudinal strain is associated with mortality and provided predictive data in normotensive septic intensive care patients.

## INTRODUCTION

Sepsis is a major global challenge associated with high mortality rates in intensive care unit (ICU) patients.^(1)^ Sepsis-induced cardiomyopathy has been identified as one of the major factors leading to death.^(2)^ Approximately 85% of septic patients admitted to the ICU have cardiac involvement, which is associated with hospital mortality.^(3)^ Two-dimensional echocardiography is a noninvasive, low-cost imaging technique for evaluating cardiac function in sepsis.^(4)^ Although left ventricular ejection fraction (LVEF) obtained from conventional echocardiography is the most commonly used method to assess left ventricle (LV) systolic function, its fundamental limitation is the inability to detect subtle cardiac dysfunction.^(5)^ Strain measurement using speckle-tracking echocardiography is a recently developed technique to assess cardiac function.^(5)^ Compared with conventional echocardiography measurement, this method is a more sensitive, reliable, and reproducible modality for assessing LV systolic function, particularly for deducing subtle LV dysfunction in the early stage of the disease.^(6,7)^ Furthermore, left ventricular global longitudinal strain (LVGLS) has been shown to be a powerful predictor of cardiovascular events and all-cause mortality.^(7)^ Accordingly, LVGLS measured by speckle-tracking echocardiography might be a good surrogate of intrinsic LV systolic function, contrary to LVEF.

There are reports investigating the association of LVGLS with outcomes in patients with sepsis.^(6,8,9)^ However, a limited number of studies address the predictive value of LVGS in normotensive septic patients.^(2)^ Therefore, our purpose was to analyze the predictive value of LVGLS in early-stage normotensive septic patients. In other words, we aimed to evaluate the predictive value of LVGLS within the first 24 hours of ICU patient admission. We hypothesized that impaired LVGLS is associated with increased mortality in normotensive septic patients in the ICU.

## METHODS

### Study design and population

This observational, prospective cohort study was performed in line with the principles of the Declaration of Helsinki. Approval was granted by the Ethics Committee of Kafkas University (May 26, 2021, No 80576354-050-99/179). Written informed consent was obtained from all patients or their legal representatives.

Consecutive adult patients with sepsis admitted to a tertiary medical ICU between June 1, 2021, and August 31, 2021, were included. Sepsis diagnosis was based on the Sepsis-3 criteria.^(10)^ Baseline clinical variables, including demographics, comorbidities, hemodynamic parameters, Glasgow Coma Scale (GCS),^(11)^ Sequential Organ Failure Assessment (SOFA),^(12)^ and Acute Physiology and Chronic Health Evaluation II (APACHE II)^(13)^ scores, were obtained and calculated within 24 hours of ICU admission. An echocardiographic examination was also performed for each subject within 24 hours of admission. Laboratory findings within the same timeframe were also analyzed.

The inclusion criteria were normotensive septic patients over the age of 18 years. The exclusion criteria were as follows: nonseptic patients and patients with septic shock; acute coronary syndrome; arrhythmias (atrial fibrillation and ventricular tachycardia); patients with metallic prosthetic mitral or aortic valves; and patients with coronavirus disease 2019 (COVID-19) infection.

### Echocardiographic measurements

**Figure 1:**
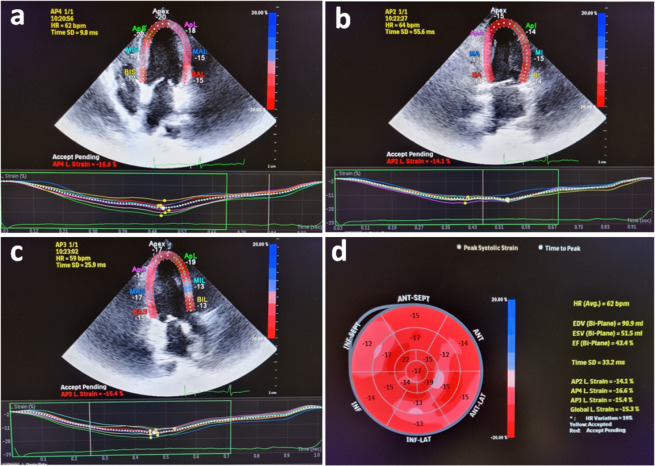
An example of left ventricular global longitudinal strain speckle tracking of a patient from the apical 4-chamber (A), 2-chamber (B), and 3-chamber (C) views. (D) The bullseye view of 17 ventricular segments from the apical 4-chamber, 3-chamber, and 2-chamber views.

Echocardiographic images were obtained using Philips Epiq7 (Philips Ultrasound, WA, United States) based on the American Society of Echocardiography (ASE) and the European Association of Cardiovascular Imaging (EACVI) guidelines.^(14)^ LV end-systolic, end-diastolic, and left atrium diameters were measured. Measurements of mitral inflow included the peak early (E-wave) and late (A-wave) diastolic filling velocities and calculation of the E/A ratio. The peak velocity of early diastolic mitral annular motion (e’) as determined by pulsed wave Doppler was measured (the average of septal and lateral) in the apical four-chamber view. Left ventricular ejection fraction was measured using the modified Simpson’s method described in the EACVI.^(14)^ Speckle-tracking analysis was performed per the consensus document of the EACVI/ASE/Industry Task Force.^(15)^ Left ventricular global longitudinal strain was analyzed by an experienced cardiologist, blinded to the outcome, using the onboard QLAB Advanced Quantification Software available in our echocardiography machine. While end-diastole was regarded as the peak R wave of the electrocardiogram, end-systole was estimated as aortic valve closure. Analysis of LV myocardial deformation was then performed from 2-dimensional grayscale loops by automatic tracking of myocardial speckles after manual selection of landmark points using apical views of the left ventricle. The region of interest was the endocardium (from the endocardial border to the myocardial midline). Left ventricular global longitudinal strain was calculated by averaging the negative peak of longitudinal strain from 17 ventricular segments from the apical 4-chamber, 3-chamber, and 2-chamber views (Figure 1). Left ventricular global longitudinal strain was expressed as a percent change (%). Negative values of LVGLS represent myocardial contractility (the less negative value, the worse LVGLS performance).

### Statistical analysis

The Statistical Package for the Social Sciences (SPSS) software version 20.0 (SPSS, Inc., Chicago, IL, United States) was used for statistical analysis. While the continuous variables were expressed as the mean values and standard deviation, categorical variables were presented as frequencies and percentages. Data were evaluated with the Kolmogorov-Smirnov test in terms of normal distribution. The independent t test was used to analyze normally distributed continuous data, and the Mann‒Whitney U test was used to analyze non-normally distributed variables. As appropriate, categorical variables were compared with the chi-squared test or Fisher’s exact test. Univariate regression analyses were performed for variables that were significantly different to identify the variables related to ICU mortality.

A multivariate logistic regression analysis, including the variables with p value < 0.05, was used to determine the independent risk factors for ICU mortality. Because our study was based on a predictive model and considered the background knowledge of the research, the cutoff value of 0.05 was chosen to better reveal clinically relevant covariates. Data are presented as odds ratios with the corresponding 95% confidence intervals (95%CI). A receiver operating characteristic (ROC) curve was used to detect the cutoff value of LVGLS in predicting ICU mortality. Additionally, Spearman correlation analysis was conducted between conventional echocardiographic parameters and LVGLS, as well as troponin value. The statistical significance level was accepted as two-tailed p values < 0.05.

## RESULTS

One hundred seventy-four patients were admitted to the ICU during the study period. Twenty-two cases were excluded following exclusion criteria. Consequently, the final study population included 152 patients [median age 62 (interquartile range - IQR, 45 - 73) years, 63.8% male]. A total of 41 (27%) patients died during hospitalization. During the ICU stay, 68% of the patients progressed to shock.

Table 1 compares the baseline demographic, laboratory, and clinical variables between survivors and non-survivors. Non-survivors were older than survivors (age [IQR], 68 years [48 - 77] *versus* 60 years [44 - 70]; p = 0.016). The percentage of patients with invasive mechanical ventilation (IMV) was higher in non-survivors than in survivors (46.3% *versus* 13,5%; p < 0.001). However, there were no significant differences in sex, hospital stay, BMI, initial vital signs, or laboratory findings.

**Table 1:** Demographic, clinical, and laboratory characteristics

	Overall (n = 152)	Survivors (n = 111)	Non-survivors (n = 41)	p value
Male	97 (63.8)	74 (66.7)	23 (56.1)	0.257
Age (years)	62 [45 - 73]	60 [44 - 70]	68 [48 - 77]	0.016
BMI	23.1 ± 4.8	22.9	23.6	0.442
Invasive mechanical ventilation	34 (22.4)	15 (13.5)	19 (46.3)	< 0.001
Admission vital signs				
MBP (mmHg)	78 [74 - 88]	79 [74 - 87]	78 [74 - 98]	0.922
Heart rate (RR/minute)	98 [72 - 110]	97 [72 - 109]	102 [93 - 110]	0.178
Laboratory				
Hemoglobin (g/dL)	12.5 [10.2 - 14.5]	13 [10.6 - 14.5]	12.3 [9.6 - 14.5]	0.593
WBC (× 10^*^3/µL)	12 [8.55 - 15]	11 [8.1 - 14.1]	12.3 [10 - 15]	0.164
Neutrophil (× 10^*^3/µL)	8.3 [5.1 - 12.15]	8.2 [5.4 - 12]	8.6 [4.5 - 12.3]	0.701
Lymphocyte (× 10^*^3/µL)	1 [1 - 2]	1 [1 - 2]	2 [1 - 3]	0.061
Platelet (× 10^*^3/µL)]	182 [145 - 216]	175 [145 - 219]	194 [156 - 215]	0.665
Hs-TnT (ng/L)	10 [7 - 17]	9 [6 - 18]	12 [8 - 16]	0.318
CRP (mg/L)	75 [23.3 - 105]	72 [18 - 98]	86 [57 - 123]	0.066
Creatinine (mg/dL)	0.90 [0.62 - 1.38]	0.92 [0.65 - 1.37]	0.75 [0.52 - 1.32]	0.248
Sodium (mEq/L)	137 [134 - 141]	138 [134 - 141]	137 [133 - 139]	0.124
Glucose (mg/dL)	108 [93 - 139]	111 [92 - 145]	105 [97 - 131]	0.519
Albumin (g/dL)	2.72 [2.25 - 3.1]	2.9 [2.27 - 3.13]	2.63 [2.3 - 3.02]	0.465
Risk scores				
GCS	10.5 [9 - 14]	12 [9 - 14]	9 [7 - 12]	< 0.001
APACHE II	15 [10 -20]	12 [9 - 19]	20 [18 - 22]	< 0.001
SOFA	9 [7 - 12]	8 [5 - 9]	12 [9 - 15]	< 0.001
Comorbidities				
Hypertension	44 (28.9)	27 (24.3)	17 (41.5)	0.039
Diabetes	36 (23.7)	27 (24.3)	9 (11)	0.833
Coronary artery disease	28 (18.4)	20 (18)	8 (19.5)	0.817
Heart failure	15 (9.9)	10 (9)	5 (12.2)	0.559
CKD (eGFR < 60mL/min/m^2^)	13 (8.6)	9 (8.1)	4 (9.8)	0.749
CVD	21 (13.8)	17 (15.3)	4 (9.8)	0.440
Source of infection				
Pulmonary	75 (49.3)	60 (54.1)	15 (36.6)	
Urinary system	17 (11.2)	9 (8.1)	8 (19.5)	
Abdominal	7 (4.6)	4 (3.6)	3 (7.3)	
Soft tissues	5 (3.3)	4 (3.6)	1 (2.4)	
Unknown	48 (31.6)	34 (30.6)	14 (34.1)	

BMI - body mass index; MBP - mean blood pressure; RR - respiratory rate; WBC - white blood count; hs-TnT - high-sensitivity troponin T; CRP - C-reactive protein; GCS - Glasgow coma scale; APACHE II - Acute Physiology and Chronic Health Evaluation II; SOFA - Sequential Organ Failure Assessment; CKD - chronic kidney disease; eGFR - estimated glomerular filtration rate; CVD - cerebrovascular disease. Results expressed as n (%), median [interquartile range] or mean ± standard deviation.

Considering comorbidities, including hypertension (28.9%), diabetes (23.7%), chronic kidney disease (8.6%), cerebrovascular disease (13.8%), coronary artery disease (18.4%), and heart failure (14.5%), only the frequency of hypertension was significantly higher in the non-survivors than in the survivors (41.5% *versus* 24.3%; p = 0.045).

When risk scores were calculated and compared between the groups, GCS was significantly lower and APACHE II and SOFA scores were significantly higher in non-survivors than in survivors (median [IQR] 9 [7 - 12] *versus* 12 [9 - 14], 20 [18 - 22] *versus* 12 [9 - 19] and 12 [9 - 15] *versus* 8 [5 - 9], respectively, the p value for all < 0.001].

Concerning echocardiographic characteristics, non-survivors had significantly less negative LVGLS (indicating worse LV function) than survivors (-15.2 [-17.2 - -12.5] *versus* -17.3 [-18.8 - -15.5]; p < 0.001). The remaining echocardiographic features were similar between the two groups (Table 2). Additionally, there was no significant relationship between LVGLS and the progression of shock (p > 0.05).

**Table 2:** Echocardiographic characteristics

	Overall (n = 152)	Survivors (n = 111)	Non-survivors (n = 41)	p value
LVEDD (mm)	51 [49 - 53]	50 [49 - 53]	52 [50 - 56]	0.058
LVESD (mm)	34 [30 - 37]	34 [32 - 37]	33 [29 - 36]	0.058
LA diameter (mm)	34 [30 - 43]	33 [29 - 42]	36 [32 - 45]	0.173
E (cm/s)	75 [69 - 77.8]	75 [69 - 77.5]	75 [70 - 77]	0.772
A (cm/s)	67 [58 - 73]	68 [58.5 - 75.5]	66 [61 - 71]	0.269
E/A ratio	1.1 [0.98 - 1.25]	1.08 [0.98 - 1.25]	1.11 [0.97 - 1.26]	0.929
e'	8 [7 - 10]	9 [8 - 10]	8 [7 - 11]	0.796
E/e' ratio	8.8 ± 1.7	8.9 ± 1.5	8.5 ± 1.9	0.237
LVEF (%)	55.04 [52 - 58.25]	56 [52 - 58.4]	54 [50 - 56]	0.164
LVGLS (%)	-16.95 [-18.38 - -14.6]	-17.3 [-18.8 - -15.5]	-15.2 [-17.2 - -12.5]	< 0.001

LVEDD - left ventricle end-diastolic diameter; LVESD - left ventricle end-systolic diameter; LA - left atrium; E - maximum flow velocity during early left ventricular diastolic filling; A - maximum flow velocity during late diastolic left ventricular filling; e' - early mitral tissue doppler velocity; LVEF - left ventricular ejection fraction; LVGLS - left ventricular global longitudinal strain. Results expressed as median [interquartile range] or mean ± standard deviation.

When univariate and multivariate analyses were performed, comprising variables that significantly differed between survivors and non-survivors (LVGLS, age, hypertension, IMV, GCS, APACHE II, and SOFA), LVGLS was found to be an independent risk factor for ICU mortality, along with IMV, GCS, APACHE II, and SOFA risk scores (OR [95%CI] 1.326 [1.038 - 1.693]; p = 0.024, 4.021 [1.073 - 15.075]; p = 0.039, 0.825 [0.696 - 0.979]; p = 0.028, 1.161 [1.065 - 1.265]; p = 0.001, 1.154 [1.032 - 1.291]; p = 0.012, respectively) (Table 3).

**Table 3:** Univariable and multivariable predictors of death

	Univariate analysis		Multivariate analysis	
	**OR (95%CI)**	**p value**	**OR (95%CI)**	**p value**
Age	1.029 (1.006 - 1.052]	0.011	0.966 (0.926 - 1.008)	0.114
Hypertension	2.204 (1.033 - 4.701)	0.041	2.323 (0.752 - 7.172)	0.143
IMV	5.527 (2.434 - 12.554]	< 0.001	4.021(1.073 - 15.075)	0.039
GCS	0.805 (0.713 - 0.910)	0.001	0.825 (0.696 - 0.979)	0.028
APACHE II	1.173 (1.097 - 1.255)	< 0.001	1.161 (1.065 - 1.265)	0.001
SOFA	1.212 (1.11 - 1.323)	< 0.001	1.154 (1.032 - 1.291)	0.012
LVGLS	1.415 (1.213 - 1.649)	< 0.001	1.326 (1.038 - 1.693)	0.024

OR - odds ratio; 95%CI - 95% confidence interval; IMV - invasive mechanical ventilation; GCS - Glasgow coma scale; APACHE II - Acute Physiology and Chronic Health Evaluation; SOFA - Sequential Organ Failure Assessment; LVGLS - left ventricular global longitudinal strain.

**Figure 2:**
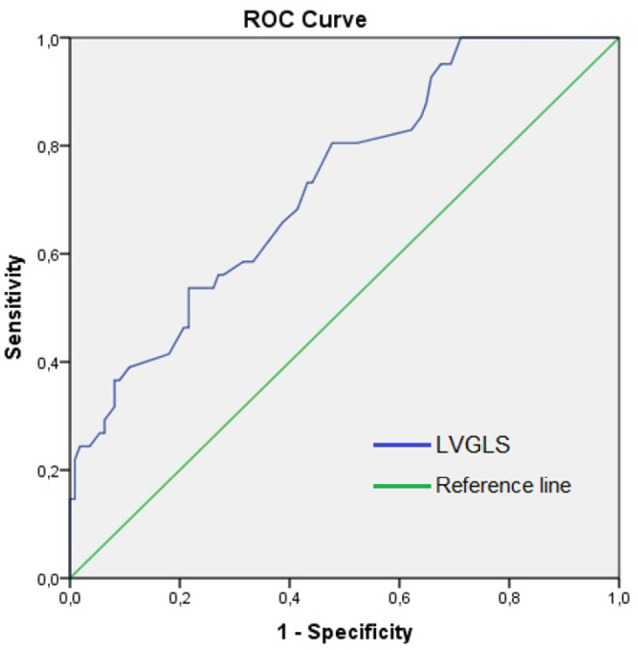
Receiver operating characteristic curve for prediction of intensive care unit mortality using the left ventricular global longitudinal strain. The area under the curve is 0.73 (cutoff: -17%, sensitivity: 73%, specificity: 57%).

A cutoff value for LVGLS was calculated using ROC analysis to predict ICU mortality (Figure 2). The area under the curve was 0.73, and the optimal cutoff value was -17 (with a sensitivity of 73% and specificity of 57%). The median LVGLS was -16.95, similar to the cutoff value. Thus, the patients were classified into two groups according to the cutoff value (GLS ≥ -17%, n = 79 and GLS < -17%, n = 73). The comparison of the variables between these two groups is summarized in table 4.

**Table 4:** Demographic, clinical, and laboratory characteristics according to left ventricular global longitudinal strain

	Overall (n = 152)	LVGLS ≥ -17% (n = 79)	LVGLS < -17% (n = 73)	p value
Death	41 (27)	31 (39.2)	10 (13.7)	< 0,001
Male	97 (63.8)	51 (64.6)	46 (63)	0.867
Age (years)	62 [45 - 72.25]	65 [55-77]	51 [41-69]	< 0.001
Intubation, mechanical ventilation	34 (22.4)	20 [25.3]	14 [19.2]	0.364
BMI	23.1 ± 4.8	23.22 ± 4.62	23 ± 5.10	0.778
Initial vital signs				
MBP (mmHg)	78 [74 - 88]	78 [73.5 - 86]	82 [75 - 89]	0.273
Heart rate (RR/minute)	98 [72 - 110]	98 [72 - 110]	100 [76 - 110]	0.740
Laboratory				
Hemoglobin (g/dL)	12.5 [10.2 - 14.5]	13 [10.15 - 14.85]	12.5 [10.2 - 13.6]	0.257
WBC (× 10^*^3/µL)	12 [8.55 - 15]	12 [9 - 14.35]	112 [8.4 - 15]	0.717
Neutrophil (× 10^*^3/µL)	8.3 [5.1 - 12.15]	8.4 [4.55 - 12]	8.2 [5.6 - 12.3]	0.453
Lymphocyte (× 10^*^3/µL)	1 [1 - 2]	1 [1 - 2]	1 [1 - 2]	0.240
Platelet (× 10^*^3/µL)	182 [145 - 216]	182 [129 - 216]	171 [151 - 215]	0.707
Hs-TnT (ng/L)	10 [7 - 17]	11 [7 - 15.5]	9 [6 - 18]	0.694
CRP (mg/L)	75 [23.25 - 105]	76 [29.5 - 101.5]	72 [18 - 109]	0.625
Creatinine (mg/dL)	0.9 [0.62 - 1.38]	1.07 [0.72 - 1.55]	0.79 [0.55 - 1.11]	0.007
Sodium (mEq/L)	137 [134 - 141]	137 [133 - 141]	137 [134 - 141]	0.712
Glucose (mg/dL)	108 [93 - 139]	108 [93 - 136.5]	108 [94 - 145]	0.893
Albumin (g/dL)	2.72 [2.25 - 3.10]	2.6 [2.21 - 3.02]	2.9 [2.4 - 3.2]	0.032
Risk scores				
GCS	10.5 [9 - 14]	10 [8 - 13.5]	12 [9 - 14]	0.221
APACHE 2	15 [10 - 20]	18 [12 - 22]	11 [9 - 18]	< 0.001
SOFA	9 [7 - 12]	9 [6-14]	8 [6 - 11]	0.156
Comorbidities				
Hypertension	44 (28.9)	23 (29.1)	21 (28.8)	0.962
Diabetes	36 (23.7)	21 (26.6)	15 (20.5)	0.382
Coronary artery disease	28 (18.4)	18 (22.8)	10 (13.7)	0.149
Heart failure	15 (9.9)	11 (13.9)	4 (5.5)	0.081
CKD (eGFR < 60mL/min/m^2^)	13 (8.6)	4 (5.1)	9 (12.3)	0.110
Previous CVD	21 (13.8)	10 (12.7)	11 (15.1)	0.667

LVGLS - left ventricular global longitudinal strain; BMI - body mass index; MBP - mean blood pressure; RR - respiratory rate; WBC - white blood count; hs-TnT - high-sensitivity troponin T; CRP - C-reactive protein; GCS - Glasgow coma scale; APACHE II - Acute Physiology and Chronic Health Evaluation II; SOFA - Sequential Organ Failure Assessment; CKD - chronic kidney disease; eGFR - estimated glomerular filtration rate; CVD - cerebrovascular disease. Results expressed as n (%), median [interquartile range] or mean ± standard deviation.

ROC - receiver operating characteristic; LVGLS - left ventricular global longitudinal strain.

**Figure 3:**
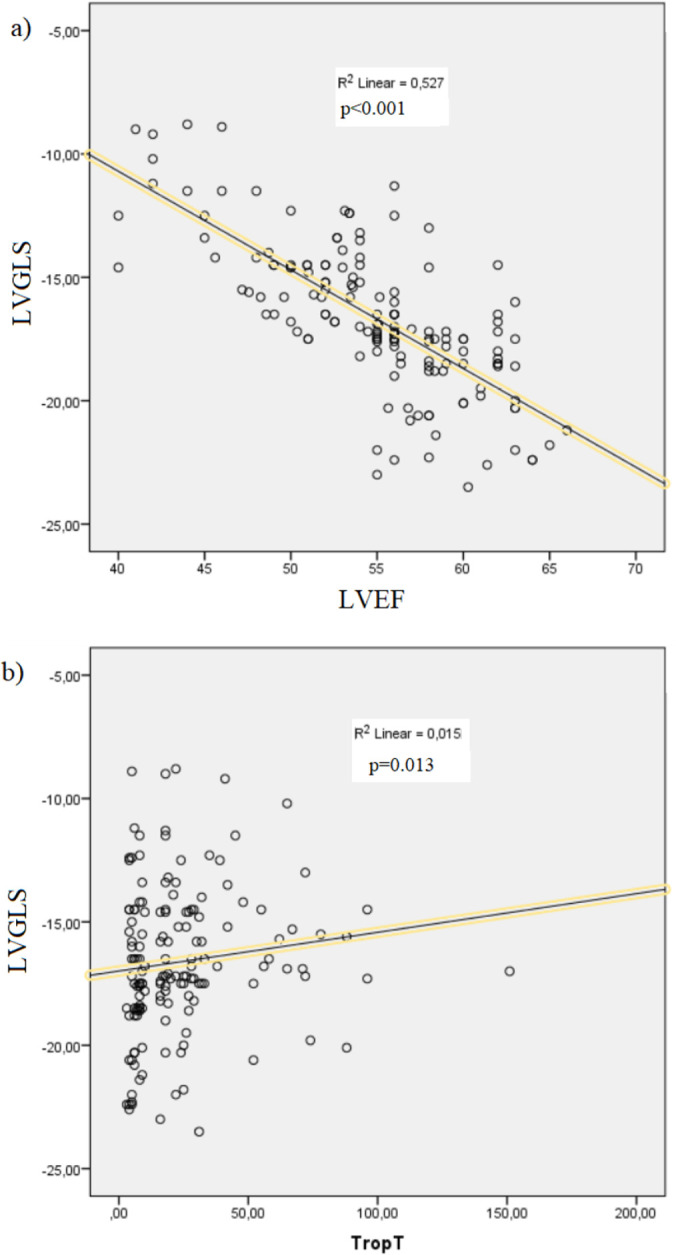
Correlation graphics between the left ventricular global longitudinal strain and left ventricular ejection fraction (A), and troponin T (B).

According to Spearman correlation analysis, LVGLS was significantly correlated with LVEF and troponin value (-0.741, p < 0.001 and 0.202, p = 0.013) (Figure 3). No significant correlation was found between the remaining conventional echocardiographic parameters and LVGLS or troponin value.

LVGLS - left ventricular global longitudinal strain; LVEF - left ventricular ejection fraction; Trop T - troponin T.

## DISCUSSION

Our study showed that impaired LVGLS was associated with a higher mortality rate in normotensive septic intensive care patients. Moreover, it was an independent predictor of ICU mortality.

Sepsis is a significant cause of mortality and morbidity and frequently associated with multiple organ failure.^(1)^ Furthermore, it substantially consumes health care resources and expenditures.^(16)^ To date, many parameters, including biochemical,^(17)^ hematological,^(18)^ demographic,^(19)^ and imaging,^(20)^ have been investigated to highlight the association between mortality and sepsis. Consistent with these studies, our study showed that older age and the proportions of hypertension and patients with IMV were significantly higher in non-survivors. However, our laboratory findings showed no significant difference, although some were associated with mortality in other reports.^(17)^

Considering LV function, increasing evidence validates the correlation between myocardial dysfunction and high mortality rates in septic patients.^(2)^ In a postmortem necropsy study on sepsis, fatal cardiovascular failure accounted for at least 35% of events, and myocardial injury was observed in more than half of the patients.^(21)^ The most commonly used method to detect LV myocardial dysfunction is LVEF.^(8)^ Nevertheless, its main limitation is the inability to detect subtle LV dysfunction, which is common in the early phase of sepsis.^(8)^ Left ventricular global longitudinal strain measured by speckle tracking echocardiography permits a better estimate of LV systolic function, particularly subtle LV systolic dysfunction.^(6)^ Numerous reports have evidenced the association between impaired LVGLS and mortality in patients with sepsis.^(2,22)^ In our study, LVGLS was significantly worse in non-survivors than in survivors, while LVEF was similar between the two groups. Similar results were established by Chang et al. in septic shock patients.^(8)^ Several pathophysiological processes in acute inflammatory states, including toxins, microvascular vasoconstriction, proinflammatory mediators, myocardial depressant factor, mitochondrial dysfunction, myocardial edema, inflammatory cell infiltration, and, consequently, myocardial injury, could lead to myocardial dysfunction.^(21,23)^ Thus, impaired LVGLS in patients with sepsis may not be surprising.

Early detection of myocardial dysfunction and prediction of the prognosis in septic patients may be crucial for facilitating prioritized treatment and more aggressive therapeutic strategies.^(7,20)^ Thus far, prognostic scoring systems such as GCS,^(11)^ APACHE II,^(13)^ and SOFA^(12)^ have been defined to predict outcomes in critically ill patients. Similarly, all three risk scores were independent predictors of ICU mortality in our study.

As the most significant outcome of our work, we found that LVGLS was an independent predictor of ICU mortality. Several studies have investigated the predictive value of LVGLS in septic intensive care patients. Palmieri et al. considered the prognostic relevance of LVEF and LVGLS in sepsis, focusing on day-7 and day-28 follow-ups.^(24)^ Similar to our study, LVEF exhibited no prognostic relevance, whereas LVGLS was correlated with mortality. Another study, including 90 septic shock patients, showed that LVGLS was an independent predictor of in-hospital mortality.^(25)^ Innocenti et al. demonstrated that reduced LV systolic function defined by LVGLS was associated with adverse shortand medium-term (day-7 and day-28 mortality, respectively) outcomes, independent of troponin level.^(26)^

Almost all the aforementioned reports investigating LVGLS in sepsis included patients with shock. The results of our study, which included septic patients without shock, may indicate that the primary mechanism of sepsis-induced LV dysfunction is due to a pathophysiological process caused by sepsis itself, rather than blood pressure alteration caused by sepsis.

## CONCLUSION

Impaired left ventricle systolic function measured by speckle-tracking echocardiography (left ventricular global longitudinal strain) provided reliable prognostic data in normotensive septic intensive care patients when performed early on. Further investigations with a broader population of critically ill septic patients, also considering the effect of blood pressure alterations, are needed.
